# Adulthood transitions in health and welfare; a literature review

**DOI:** 10.1002/nop2.136

**Published:** 2018-03-06

**Authors:** Berit Munck, Anita Björklund, Inger Jansson, Kristina Lundberg, Petra Wagman

**Affiliations:** ^1^ School of Health and Welfare ADULT research group Jönköping University Jönköping Sweden; ^2^ Faculty of Caring science, Work Life and Social Welfare Pre Hospen ‐ Centre for Prehospital Research University of Borås Borås Sweden

**Keywords:** developmental transition, health illness transition, life style transition, literature review, nurse education, organizational transition, situational transition

## Abstract

**Aim:**

The aim of the literature review was to describe how adulthood transition is used in health and welfare.

**Design:**

A qualitative design with a deductive approach were used.

**Methods:**

As material, 283 articles published in scientific journals, between 2011–August 2013, were selected. The search was conducted August 2013. The data were analysed and sorted in a categorization matrix.

**Results:**

Transition was identified as a process mainly related to the four types previously identified; developmental, situational, health‐illness and organizational transitions. Another one transition was also identified, lifestyle transition.

## 1 INTRODUCTION

Transition is a common concept in health and welfare education, as well as in practice. Health professionals often meet people during transitional periods regarding health and illness (Meleis, 1985). In nurse education, it is used to describe people's changes in health status, role relationships or expectations.

Chick and Meleis (1986) initiated the development of a theory of transition in nursing by examining the structure and function of it. They defined transition as a passage from one life phase, condition, or status to another, including elements of process, time span and perception. The process of transition is associated with a sense of movement or development, or an adaption to a new situation. The transition process includes both the situation that is its cause and the person's responses to these changes. The time span comprises both a beginning and an end, not occurring simultaneously and extends from the first expectance of transition until stability has been achieved and can be short or long. Finally, perceptions reflect differences in how transition events are experienced and people's reactions and responses to them (Chick & Meleis, 1986).

Later, Meleis, Sawyer, Im, Messias, and Schumacher (2000) identified several properties of transitional experiences, including the following: awareness, engagement, change and difference, time span and critical points and events. Awareness is associated with how a person perceives, has knowledge of and recognizes the experience of transition. According to Chick and Meleis (1986), awareness of transitions is necessary, otherwise there is no transition. However, Meleis et al. (2000) argued that awareness is an important property of transition, but its absence does not exclude transition from occurring. Furthermore, while Chick and Meleis (1986) proposed that transition essentially is a positive experience other claim that transition may also lead to deterioration (Parkes, 1971; Schlossberg, 1981).

Engagement is associated with the level of awareness in relation to the transition and cannot occur if a person is unaware of the transition. Change and difference are other properties of transitions; all transitions include a change, but not all changes are related to transition. Difference implies the perception of being different or seeing the environment in different ways. Regarding time span, Bridges (1996) defined this as including the first perceptions of changes, through a period of instability to an eventual ending and a new beginning and period of stability (Bridges, 1996). However, Meleis et al. argue that it is difficult or impossible to put boundaries on the time span of the transition experiences because some are never‐ending processes (Meleis et al., 2000). Furthermore, some transitions are associated with critical turning points and events, such as birth, death or the diagnosis of an illness. In other transitions, though, specific marker events are not that obvious (Meleis et al., 2000).

Transition was initially divided into three types: “developmental”, “situational” and “health‐illness transition's” (Meleis, 1985), but subsequently an additional type was identified, “organizational transition's” (Schumacher & Meleis, 1994). A developmental transition can be the transition from childhood to adolescence or from adulthood to mature adulthood. The situational transition may constitute an addition or a subtraction of persons, which requires a redefinition of roles, such as loss of a family member through divorce or death. The health‐illness transition is for example a movement from well state to illness, or a movement from critical care and back to the community and vice versa (Meleis, 1985; Schumacher & Meleis, 1994). Finally, organizational transitions represent changes in the environment related to social, political or economic changes, such as the adoption of new policies, changes in leadership, role changes, implementation of new models or introduction of new technology (Schumacher & Meleis, 1994).

In healthcare contexts, transitions may imply problematic periods for individuals as well as caregivers and occur all‐through the life span. Individuals’ developmental changes from child to adolescent in combination with the change from pediatric to adult health care are critical transitional situations and substantial gaps between healthcare providers have been identified in e.g., diabetes care (Hilliard et al., 2014) as well as inflammatory bowel disease care (Maddux, Ricks, & Bass, 2017). Organizational transitions of vulnerable people from different healthcare contexts in combination with changes of life situations i.e., developmental transitions, are precarious situations and put demands on healthcare professionals. A need for education in transitional situations has been identified in several studies such as the transition from adolescent to adult medical care for person with autism spectrum disorder (Rogers & Zeni, 2015) and for patients in transition into end‐of‐life care (Martinsson, Heedman, Eriksson, Tavelin, & Axelsson, 2016)). The transition to parenthood may be an overwhelming life event where healthcare professionals can benefit education and thus facilitate this process (Barimani, Vikström, Rosander, Forslund Frykedal, & Berlin, 2017).

Taken together, transition is a frequently used concept in health and welfare education and practice, appearing in various contexts but further knowledge is needed about its current use. Hence, the aim of this literature review was to describe how adulthood transition is used in health and welfare.

## 2 THE METHOD

### 2.1 Design

This literature review used a qualitative design with a deductive approach (Elo & Kyngäs, 2008) and articles published in scientific journals as data.

### 2.2 Search methods

Articles published in scientific journals were used as data material. The search for articles was conducted August 29 in 2013 using the following data bases combined: Amed, Cinahl, Dentistry and Oral Sciences, Eric, Medline and Socindex. The search string used was transition* AND (welfare OR MH health OR TI health) NOT (Health Transition OR Health Services OR Foreign Nurses OR Collaboration OR Community Networks OR Students OR Volunteer Workers OR Health Care Delivery OR New Graduate Nurses OR Preceptorship).

The criteria for inclusion of articles were that they were published between 2011–August 2013: in English; had transition in the heading, abstract, or as a key word; and were related to transition in adult life, from child to adult, or from adult to old age. Exclusion criteria were articles containing conference proceedings, editorials, or letters to the editor.

### 2.3 Search outcome

**Figure 1:**
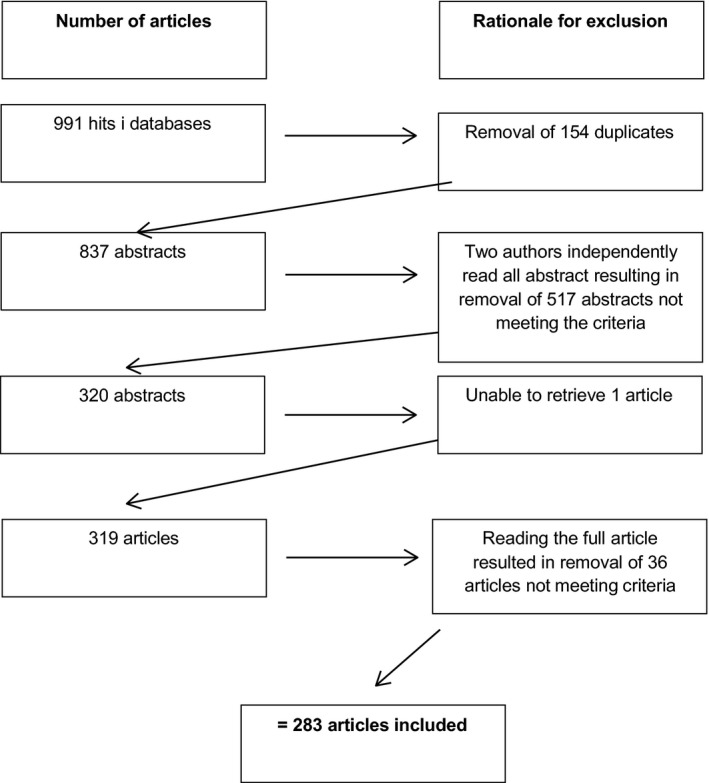
The process of data gathering

The search resulted in 991 hits and the selection process, ending up in the 283 articles, is shown in Figure 1.

### 2.4 Quality appraisal

The present literature review aims to describe how adulthood transition is used in the area of health and welfare. It focuses on the concept and therefore considered relevant to include articles varying in design to get information about its use from a broader perspective. Thus, it was not considered relevant to assess the quality of the articles included beyond controlling that they matched the inclusion criteria chosen.

### 2.5 Analysis

The data analysis began with the authors piloting ten articles independently. It resulted in a categorization matrix (Elo & Kyngäs, 2008) comprising a column for each of the four types of transition: developmental, situational, health‐illness and organizational transition (Schumacher & Meleis, 1994) and a column marked other.

### 2.6 Data abstraction

Thereafter, all relevant data from the articles was extracted and sorted into the relevant column in the matrix depending on which type of transition it was considered to belong to. The authors met regularly to discuss the analysis and findings. During these meetings, consensus was sought regarding the various types of transitions. The data not considered as belonging to any of the four types (other) were studied more in detail, using an inductive approach, to analyse its content.

### 2.7 Ethics

Patient consent and ethical approval was not required for the literature review.

## 3 RESULTS

The analysis showed that most articles could be related to the four types of transition previously identified by Schumacher and Meleis (1994) developmental, situational, health‐illness and organizational. The content in the group of articles considered as belonging to “other”, i.e., not to any of the four previously identified types of transition, was found to embrace: physical activity, eating habits, tobacco use, drug use and risk tendency. The authors concluded that they all represented *lifestyle matters* in one way or another, good or bad, ending up in the identification of a “new” type of transition, called “lifestyle transitions”.

### 3.1 The types of transitions previously identified: Developmental, situational, health‐illness and organizational

All four types of transition identified by Schumacher and Meleis (1994) were seen in the results. Developmental transition (Meleis, 1985; Schumacher & Meleis, 1994) in our findings concerned the development from a biological perspective, such as menopausal (Greendale, Ishii, Huang, & Karlamangla, 2013; Mitchell & Woods, 2013). It also contained life‐cycle transitions, such as those from childhood to adulthood (Lee, Courtney, & Hook, 2012; Serracant, 2012) or from adolescence to adulthood (Allen & Williams, 2012) and life role transitions, such as the transition to parenthood (Behague, Goncalves, Gigante, & Kirkwood, 2012; Wardrop & Popadiuk, 2013) or grandparenthood (McKinley, Brown, & Caldwell, 2012; Taubman ‐ Ben‐Ari, Findler, & Shlomo, 2013). Yet another type of developemental transition was the gender transition (Brown et al., 2013; Macdonnell & Grigorovich, 2012). A “situational transition” (Meleis, 1985; Schumacher & Meleis, 1994) was described as a transition from one state to another, such as an employment transition (Butterworth et al., 2011; Cook, 2012), transition to retirement (Calvo, Sarkisian, & Tamborini, 2013; Oksanen et al., 2011) or marriage (Hewitt, Turrell, & Giskes, 2012; Robards, Evandrou, Falkingham, & Vlachantoni, 2012) and educational transitions (Perry, 2012; Salmela‐Aro, 2012). The “health‐illness transition” type (Meleis, 1985; Schumacher & Meleis, 1994) included care transitions such as moving from one care setting to another (Bryant, Young, Cesario, & Binder, 2011; Kuchenbuch, Chemaly, Chiron, Dulac, & Nabbout, 2013; LaRosa, Glah, Baluarte, & Meyers, 2011) as well as transitons in health status from health to illness and vice versa (Lally & Underhill, 2012; Söderlund, 2011). “Organizational transitions” represented transitions in social, political or economic environments (Schumacher & Meleis, 1994) characterized as transitions in meso and macro levels, such as programs and political and societal structures (Bobić, 2012; Christensen, 2013; Perez, Blandon, Persson, Pena, & Kallestal, 2012).

### 3.2 The new type of transition identified – lifestyle transition

The new type of transition identified, not previously mentioned by Schumacher and Meleis (1994) was the “lifestyle transition” – characterized as behavioural changes regarding lifestyle matters. Lifestyle transitions included: physical activity, eating habits, tobacco use, drug use and risk tendency.

Physical activity referred to participation in fitness related activities (Patel et al., 2011), such as the transition from using a car to bicycle (Hartog, Boogaard, Nijland, & Hoek, 2011). Eating habits included transitions from overweight to obesity (Kouvonen et al., 2011), prevention of being overweight by transitioning to a healthier lifestyle (Renes, Mutsaers, & van Woerkum, 2012), as well as the promotion of healthy behaviours among overweight postpartum women (Boothe, Brouwer, Carter‐Edwards, & Ostbye, 2011). Another aspect regarding eating habits concerned the societal level and was manifested among Inuites and their transition from traditional food, such as seal, to market and junk food (Bjerregaard & Mulvad, 2012; Zhou, Kubow, & Egeland, 2011).

Transitions regarding tobacco use were found in relation to nicotine dependence (Kushner, Menary, Maurer, & Thuras, 2012) and increased nicotine use (Khaled, Bulloch, Williams, Lavorato, & Patten, 2011). Drug use transitions, including alcohol use, could lead to either disorders or remission (Silveira et al., 2011). Other drug use transitions identified were the transitions from opium to heroin and drug injection (Dolan et al., 2011) and from long lasting cannabis use to heavier use (Hyshka, 2013). Finally, the risk tendency transition was identified, which implies the transition from a risky lifestyle to less harmful behaviour (Munoz‐Laboy et al., 2012).

The lifestyle matters identified had either a positive direction, from unhealthy to healthier behaviours (Boothe et al., 2011; Hartog et al., 2011; Munoz‐Laboy et al., 2012; Patel et al., 2011; Renes et al., 2012), or a negative direction from healthy to unhealthier behaviours (Bjerregaard & Mulvad, 2012; Khaled et al., 2011; Kushner et al., 2012; Zhou et al., 2011). Positive behavioural changes included the transition from unhealthy behaviour to healthier behaviour, including the promotion of healthy behaviours among overweight postpartum women to prevent being overweight (Boothe et al., 2011; Renes et al., 2012), increased physical activity (Hartog et al., 2011; Patel et al., 2011) and the change from a risky lifestyle to less harmful behaviour (Munoz‐Laboy et al., 2012). A negative behavioural change occurred regarding dietary, when the selection of food changed from healthy food habits to more market food and junk food (Bjerregaard & Mulvad, 2012; Zhou et al., 2011). Lifestyle transitions could also be characterized as amplifying a behaviour in a negative way, such as a transition from unhealthy behaviour to even more destructive behaviour e.g., as exemplified in increasing nicotine use (Khaled et al., 2011), heavier drug abuse (Dolan et al., 2011; Hyshka, 2013; Khaled et al., 2011) and the transition from overweight to obesity (Kouvonen et al., 2011).

## 4 DISCUSSION

This literature review aimed to describe how adulthood transition is used in the area of health and welfare. The results demonstrated that transition remains a frequently used concept, as reflected in the great number of articles included. Our results also show that the concept mainly is used in line with the types previously identified (Meleis, 1985; Schumacher & Meleis, 1994). However, the identification of a fifth type of transition – lifestyle transition, indicates that the concept has developed over time. Perhaps, this finding also indicates changes in society as many years have passed since the previous types were identified.

After the year 2000, it has been an emerging emphasis on the individual's responsibility for maintaining health through lifestyle choices (Ahola‐Launonen, 2015). In the life style transition an additional concept, nutrition transition, has emerged (Lindsay et al., 2009; Traissac et al., 2015) and it refers to less physical activity in combination with increased consumption of energy‐dense food and eating behaviours (Pham, Worsley, Lawrence, & Marshall, 2017; Shaikh et al., 2017). Among the identified properties of the experience of transition (Meleis et al., 2000) awareness is of certain interest. The necessity of being aware of one's own transition could be accentuated. Transition has been described as an inner reorientation and transformation. This means that a person needs to acknowledge his change before the transition can begin (Kralik, Visentin, & van Loon, 2006). Furthermore, according to Meleis et al. (2000) a lack of awareness signifies that an individual may not be ready for a transition. However, it could be questioned whether awareness about changes is a prerequisite for lifestyle transitions. Instead some of them may be long‐lasting processes and appear without the individuals being aware of them. For instance, the transition from being overweight to being obese is probably not thought of as a transition by the individual. Rather it is an ongoing process and possibly not based on a conscious choice.

The importance of awareness of transitions can manifest itself in various ways. On an individual level it can be exemplified as transitions of unrepresented patients who lack decision‐making capacity and are in need of an advocate, which is a problem in many healthcare situations (Abdool et al., 2016). On a societal level, awareness of nutrition transition is of great importance since transition can emerge in different directions. For instance, overweight and undernutrition may co‐exist in the same population and put demands on awareness of transitional issues among healthcare professionals (Lindsay et al., 2009; Pham et al., 2017) which may be an important issue for healthcare education. Taken together, identification of lifestyle transition as an additional type of transitions as well as the possibility that individuals may not be aware of some life style transitions contribute to increased knowledge of the current use of transition. Furthermore, this finding highlights the need of healthcare professionals and educators recognizing this new type of transition and how to promote healthy lifestyle transitions and prevent unhealthy ones. The appearance of lifestyle transitions in the results may also be an indicator of a less accepting view of the right to health care for everyone (Stegeman, Willems, Dekker, & Bossuyt, 2014). As the possibilities to treat diseases and health‐related problems have increased, this has also led to more use of health care and consequently to greater costs (Stegeman et al., 2014).

### 4.1 Methodological considerations

The discovery of a new type of transition, lifestyle transition, could be regarded as our main finding, but, it cannot be ruled out that something might have been missed. Sometimes, it was also difficult to decide which type a certain transition was and this may reflect the time that has passed since they were originally identified (Schumacher & Meleis, 1994). Situational transition was perceived as the category most problematic to identify and define. For instance, there was a problem with separating developmental transitions from situational transitions. It was not always clear if a transition occurred because of situational circumstances or an individual's development. On the contrary, the health‐illness transition was not a problematic category to identify, potentially reflecting the authors’ backgrounds in health and welfare.

The present analysis also included many articles from different areas in health and welfare which is a strength considering the possibility to include “all current use”. Additionally, the authors’ professional experiences from different fields of health care also contributed to secure rigor in the literature review.

### 4.2 Limitations

There are also limitations with the present study. Firstly, solely including articles from the years 2011–2013 could be considered a limitation; but was necessary regarding the number of articles containing the concept. The decision not to include articles published later than 2013 is, potentially, a more severe limitation, but a product of a long process with analysing the results. Another option would have been to make new searches with an extended timeline. However, this would imply another limitation in the literature search since the publication date and the time update of a database is not necessarily the same. Verification of this, together with new analyses, would be very time consuming and we decided not to do that. Another potential limitation, but also an asset concerning inter‐reliability, was the fact that there were several researchers included in the analysis.

## 5 CONCLUSION

This literature review aimed to describe how the concept transition is used in the area of health and welfare in adulthood. The results showed that most of the uses of transition belong to the previous identified types: developmental, situational, health‐illness and organizational transitions. Additionally, another type of transition was also identified, the lifestyle transition. Awareness of life style transitions and their complexity is of importance on a societal level as well as among healthcare professionals to support individuals to manage behavioural changes regarding lifestyle matters.

## CONFLICT OF INTERESTS

No conflict of interest has been declared by the authors.
